# P03-020 - A novel 15-HPGD mutation in pachydermoperiostosis

**DOI:** 10.1186/1546-0096-11-S1-A218

**Published:** 2013-11-08

**Authors:** E Erken, Ç Köroğlu, F Yıldız, HT Özer, B Gülek, A Tolun

**Affiliations:** 1 Rheumatology-Immunology, Cukurova University Faculty of Medicine, Adana, Turkey; 2 Molecular Biology and Genetics, Boğaziçi University, Istanbul, Turkey; 3 Radiology, Adana Numune Hospital, Adana, Turkey

## Introduction

Autosomal recessive primary hypertrophic osteoathropathy (PHO), also known as pachydermoperiostosis (PDP), is a rare genetic disease characterized by clubbing of the fingers, arthritis, periostosis and pachydermia and results from mutations in 15-hydroxyprostaglandin dehydrogenase (HPGD)*.* Recessive mutations in 15-hydroxyprostaglandin dehydrogenase in PHO subjects. has been identified since 2008. Both homozygous and compound heterozygous mutations in HPGD have been reported. Homozygous patients had increased sustained prostaglandin E2 levels and prominent clinical and biochemical PHO.

## Objectives

To perform clinical investigations, to attempt medical treatment, and to find the HPGD mutation, the gene responsible for the disease, in a 22-year old Turkish male and his 23-year old sister afflicted with primary hypertrophic osteoarthropathy as well 14 members of their family.

## Methods

In combination with NSAIDs and colchicine, sulfasalazine was commenced to both of them, and methotrexate was added to the treatment regimen of the female patient at the end of the first year. All seven exons of gene *HPGD* including 5’ and 3’ UTRs were analyzed by direct sequencing. After the identification of the mutation, a primer pair was designed for the PCR amplification of a 152 bp-region harbouring the mutation site. Mutational analysis was repeated via high-resolution melting curve analysis performed on LightCycler 480 system, along with samples from 136 control individuals.

## Results

A homozygous 2-bp deletion (c.310_311delCT or p.L104AfsX3) was identified. Eight relatives carrying the mutation in the heterozygous state were examined and none was found affected. The patients were found typical PHO. Ultrasonographic examination of the joints revealed synovitis and inflammation by B mode and power doppler ultrasonography. One of the patients had emphysema in addition to other findings reported as associated with PHO. Joint symptoms responded to Sulfasalazine treatment in both patients. However, after addition of methotrexate, the female patient had better remission.

## Conclusion

Novel p.L104AfsX3 in *HPGD* underlies PHO in the family. Emphysema is an additional clinical finding associated with PHO. Sulfasalazin as well as methotrexate can be used for the treatment of joint symptoms.

## Competing interests

None Declared.

